# CHA_2_DS_2_-VA Score Can Be Used to Predict In-Hospital Mortality in Patients with Acute Aortic Dissection

**DOI:** 10.21470/1678-9741-2025-0303

**Published:** 2025-12-10

**Authors:** Mustafa Lutfullah Ardic, Hazar Harbalioğlu, Nasir Ali Tokmak, Hacı Ali Ucak, Hilmi Erdem Sumbul, Fadime Koca, Hasan Koca, Abdullah Eren Cetin, Mevlut Koc

**Affiliations:** 1 Department of Cardiology, University of Health Sciences, Adana Health Practice and Research Center, Adana, Turkey.; 2 Department of Cardiovascular Surgery, Adana Health Practice and Research Center, Adana, Turkey.; 3 Department of Internal Medicine, University of Health Sciences, Adana Health Practice and Research Center, Adana, Turkey.; 4 Department of Cardiology, Cukurova State Hospital, Adana, Turkey.; 5 Department of Cardiology, 25 Aralik State Hospital, Gaziantep, Turkey.

**Keywords:** Acute Aortic Dissection, Mortality, CHA_2_DS_2_-VA Score, hs-CRP

## Abstract

**Introduction:**

he CHA_2_DS_2_-VA score, which is used to determine the risk of thromboembolism in patients with atrial fibrillation, has been shown to be a predictor of mortality in many cardiovascular diseases. However, there is no data in the literature on the effect of CHA_2_DS_2_-VA score on in-hospital mortality in patients with acute aortic dissection (AAD). We aimed to determine the effect of CHA_2_DS_2_-VA score on in-hospital mortality in patients with AAD.

**Methods:**

This retrospective cohort study included 113 patients (89 males, 24 females, age 58.7±10.5 years) who underwent surgical treatment for AAD. CHA_2_DS_2_-VA scores were calculated. All cases of in-hospital mortality during the follow-up period were identified and recorded. Patients were grouped as with and without mortality.

**Results:**

Among patients with AAD, in-hospital mortality was observed in 30 cases (27.5%). Mortality rates in patients with CHA_2_DS_2_-VA 1, 2, 3 and ≥ 4 were 7%, 13%, 27%, and 53%, respectively. Age, CHA_2_DS_2_-VA score, and high-sensitivity C-reactive protein serum level independently determined the patients with mortality, and each one unit increase in these parameters predicted 11.3%, 2.19-fold, and 3.2% mortality increase, respectively. In receiver operating characteristic analysis, when the cutoff value of CHA_2_DS_2_-VA score was taken as 3, it was found to determine the development of mortality due to AAD with 80.5% sensitivity and 78.6% specificity.

**Conclusion:**

CHA_2_DS_2_-VA score is independently associated with the development of in-hospital mortality in patients with AAD. According to the findings of our study, the CHA_2_DS_2_-VA score may serve as a prognostic marker in patients with AAD.

## INTRODUCTION

**Table:** 

Abbreviations, Acronyms & Symbols				
AAD	= Acute aortic dissection		eGFR	= Estimated glomerular filtration rate
ACEi	= Angiotensin-converting enzyme inhibitors		hs-CRP	= High-sensitivity C-reactive protein
AF	= Atrial fibrillation		HF	= Heart failure
ARB	= Angiotensin receptor blockers		HT	= Hypertension
ARNI	= Angiotensin receptor-neprilysin inhibitor		LVEF	= Left ventricular ejection fraction
CKD	= Chronic kidney disease		NT-proBNP	= N-terminal pro-brain natriuretic peptide
CV	= Cardiovascular		ROC	= Receiver operating characteristic
DM	= Diabetes mellitus			

The CHA_2_DS_2_-VA score is a scoring system used to determine the risk of thromboembolism in patients with atrial fibrillation (AF)^[1]^. This scoring is calculated based on the following factors: (1) C = congestive heart failure (HF) (1 point), (2) H = hypertension (HT) (1 point), (3) A = age ≥ 75 (2 points), (4) D = diabetes mellitus (DM) (1 point), S = history of stroke (2 points), (6) V = history of vascular disease (1 point), and (7) A = age 65-74 (1 point). This scoring previously included female sex (CHA_2_DS_2_-VASc), and this risk factor was removed in the latest AF guidelines^[1]^. Studies conducted in the last 10 years have shown that the CHA_2_DS_2_-VASc score is a predictor of mortality in many cardiovascular (CV) diseases^[2-14]^. These CV diseases include AF^[2]^, acute coronary syndromes with and without ST elevation^[3-5]^, HF^[6]^, aortic stenosis^[7]^, peripheral vascular disease^[8,9]^, coronary bypass surgery^[10]^, pulmonary embolism^[11]^, stroke^[12]^, HT^[13]^, and infective endocarditis^[14]^. The CHA_2_DS_2_-VASc score has also been shown to be a predictor of mortality in clinical conditions other than CV disease, such as chronic kidney disease (CKD), coronavirus disease 2019, and chronic obstructive pulmonary disease^[15-17]^.

Acute aortic dissection (AAD) is an important CV disease with a frequency of three to six per 100000 in the general population, with a high mortality risk^[18]^. In-hospital mortality is approximately 36 - 48%, with a 1 - 2% increase in mortality per hour until operation^[19]^. Except for DM, all risk factors included in the CHA_2_DS_2_-VA score have been shown to be associated with mortality in these patients^[13,18-25]^. There are conflicting results regarding the increase in mortality in patients with DM and AAD^[21,26,27]^. However, to the best of our knowledge there is no data in the literature on the effect of CHA_2_DS_2_-VA score on in-hospital mortality in patients with AAD. We considered that CHA_2_DS_2_-VA score, an objective scoring system, could be used as a prognostic marker in AAD patients who have high mortality and morbidity.

Therefore, the aim of our study was to evaluate the effect of the CHA_2_DS_2_-VA score on in-hospital mortality in patients with AAD.

## METHODS

### Study Population

In this retrospective cohort study, 512 patients registered in the system with a diagnosis of AAD in our hospital between May 2017 and May 2025 were screened. Patients in whom emergency surgery was decided with the latest guideline recommendation and operated under emergency conditions were identified^[18,20]^. To determine the number of patients to be included in the study, a power analysis was performed considering previous studies and their results (80% power and *P* < 0.05). After this analysis, it was seen that approximately 90 patients were sufficient to be included in the study. In the study, the records of patients who underwent surgery for AAD were reviewed. Patients ≤ 18 years of age, with subacute (15 - 90 days) and chronic (> 90 days) aortic dissection, pregnant and < 3 months post-pregnancy, with chronic inflammatory disease, dialysis treatment and known end-stage renal failure, and with active cancer were excluded. The necessary permissions for the study were obtained from the ethics committee of our regional hospital (Health Sciences University Adana City Training and Research Hospital Ethics Committee on 08.05.2025 with decision number 485). The study was conducted in accordance with the Declaration of 1964 Helsinki.

After the patients were included in the study, their preoperative anamnesis and physical examination were evaluated. Demographic parameters such as age, sex, HT, DM, hyperlipidemia, smoking, previous history of cerebrovascular disease, and presence of CKD were recorded. Systolic and diastolic blood pressures and pulse parameters were obtained. The CHA_2_DS_2_-VA score was calculated by questioning the previously mentioned parameters individually, as described in the European Heart Association 2024 AF guideline^[1]^. Patients' active medical treatment was recorded. Complete blood count, blood glucose, serum blood urea nitrogen, creatinine, estimated glomerular filtration rate (eGFR), thyroid stimulating hormone, albumin, total proteins, high-sensitivity C-reactive protein (hs-CRP), and sodium were measured using automated devices (Abbott Aeroset, MN, USA) and an acceptable kit (Abbott), and potassium, uric acid, total cholesterol, low-density lipoprotein cholesterol, high-density lipoprotein cholesterol, triglycerides, alanine aminotransferase, aspartate aminotransferase, calcium, high-sensitivity troponin T, and N-terminal pro-brain natriuretic peptide (NT-proBNP) levels were also measured. Left ventricular ejection fraction (LVEF) was then calculated automatically according to Simpson's rule^[28]^. The diagnoses of malperfusion and shock were established based on a combination of patient history, physical examination findings, laboratory values, and imaging results. Malperfusion was defined as the presence of absent peripheral pulses accompanied by pain, motor or sensory deficits in the extremities, neurological deficits, signs of renal ischemia, and laboratory or imaging findings indicative of abnormal perfusion. Cardiogenic shock was defined as a systolic blood pressure of ≤ 90 mmHg for 30 minutes or longer, in the presence of both clinical and biochemical evidence of tissue hypoperfusion.

### Diagnosis of Acute Aortic Dissection

The diagnosis of AAD was made following the algorithm described in the guidelines^[18,20]^. Firstly, echocardiography was performed in addition to routine evaluations in patients presenting to the emergency department with chest pain. Patients with suspected AAD were subjected to aortic evaluation with contrast-enhanced multidetector computed tomography. As a result of this evaluation, emergency surgery was decided for patients with Stanford Type A dissection, patients with Stanford Type B dissection who could not undergo interventional treatment, and patients with complications such as i) persistent pain and dissection, ii) periaortic hematoma, mediastinal hematoma, or rupture, iii) peripheral ischemia^[18,20]^. The follow-up period of the patients was considered as the period from the day of hospitalization to the day of discharge.

### Statistical Analysis

All analyses were performed using IBM SPSS Statistics for Windows, version 23.0 (IBM Corp., Armonk, N.Y., USA). Continuous variables in group data were expressed as mean ± standard deviation. Categorical variables were expressed as number and percentage. The "Kolmogorov-Smirnov" test was used to assess whether the distribution of continuous variables is normal. In the comparison of countable parameters between two groups, Student’s *t*-test and Mann-Whitney U test were used according to normal and non-normal distribution, respectively. The chi-square test was used to compare categorical data. Fisher's exact test was used to determine whether mortality rates differed according to CHA_2_DS_2_-VA score. All parameters that were significant in the univariable analysis (*P* < 0.05) were evaluated by multivariable logistic regression analysis to identify patients with mortality. Receiver operating characteristic (ROC) analysis was performed to determine the cutoff value of CHA_2_DS_2_-VA score, which is independently predictive of mortality. Statistical significance was defined as a *P*-value < 0.05 for all comparisons.

## RESULTS

In this study, 113 patients (89 males, 24 females, age 58.7 ± 10.5 years) operated for AAD were included. In-hospital follow-up of 11.9 ± 7.9 days revealed mortality in 30 (27.5%) patients. Patients were grouped as with and without mortality. Parameters associated with mortality were determined.

### Demographic and Clinical Data of Patients with and without Mortality

Demographic and clinical data of patients with and without mortality are shown in Table 1. Age, frequency of HT, previous history of cerebrovascular event, frequency of CKD, and heart rate were found to be significantly higher in patients with mortality compared to patients without mortality. The systolic blood pressure of the mortality group was significantly lower than the non-mortality group. Target organ malperfusion and shock status were significantly higher in patients with mortality due to AAD. There were no patients with a CHA_2_DS_2_-VA score of 0, as all patients included in the study had vascular disease. When all patients were evaluated, the mean CHA_2_DS_2_-VA score of the patients included in the study was 2.41 ± 1.14. CHA_2_DS_2_-VA score was significantly higher in patients with mortality compared to those without mortality. When mortality rates were analyzed according to CHA_2_DS_2_-VA score in all patients, it was determined that mortality rates increased with the increase in CHA_2_DS_2_-VA score. Mortality rates were 7% in patients with CHA_2_DS_2_-VA = 1, 13% in patients with CHA_2_DS_2_-VA = 2, 27% in patients with CHA_2_DS_2_-VA = 3, and 53% in patients with CHA_2_DS_2_-VA ≥ 4. Statistical analysis revealed a statistically significant relationship between the groups in terms of mortality rates. Other demographic and clinical data were similar between the two groups (Table 1).

**Table 1:** Demographic and clinical data of patient groups with and without mortality due to acute aortic dissection.

Variables	Mortality (+)	Mortality (-)	*P*-value
	n = 30	n = 83	
Age (years)	68.4 ± 8.4	55.1 ± 8.9	**< 0.001**
Sex (male), n	24 (80%)	65 (78%)	0.536
Body mass index (kg/m2)	24.6 ± 3.1	24.9 ± 3.9	0.238
Hypertension, n (%)	24 (80%)	49 (59%)	**0.019**
Diabetes mellitus, n (%)	3 (10%)	9 (11%)	0.902
Smoking, n (%)	15 (50%)	50 (60%)	0.224
Hypercholesterolemia, n (%)	8 (27%)	41 (49%)	0.504
Previous cerebrovascular accident, n (%)	6 (30%)	3 (4%)	**0.002**
Chronic kidney disease, n (%)	14 (47%)	14 (17%)	**0.002**
Systolic blood pressure (mmHg)	110 ± 29	126 ± 20	**0.007**
Diastolic blood pressure (mmHg)	71 ± 20	77 ± 18	0.094
Heart rate (beat/min)	96 ± 18	84 ± 11	**0.002**
Cardiac tamponade, n (%)	1 (3%)	5 (6%)	0.494
Malperfusion, n (%)	9 (30%)	6 (7%)	**0.004**
Shock, n (%)	8 (27%)	7 (21%)	**0.006**
CHA_2_DS_2_-VA score group			**< 0.001**
≥ 4, n	16	3	
3, n	8	14	
2, n	4	43	
1, n	2	22	
CHA_2_DS_2_-VA score	3.53 ± 1.22	2.00 ± 0.78	< 0.001

### Laboratory Medical Treatment Data of Patients with and without Mortality

Laboratory and medical treatment data of patients with and without mortality are shown in Tables 2 and 3. Among these parameters, serum levels of white blood cell count, creatinine, blood urea nitrogen, eGFR, hs-CRP, and NT-proBNP were significantly higher in patients with mortality compared to those without mortality. Low-density lipoprotein cholesterol and LVEF values were significantly lower in patients with mortality. In addition, renin angiotensin aldosterone system blocker and statin use were significantly lower in patients with mortality. Other laboratory and medical treatment data were similar between the two groups (Tables 2 and 3).

**Table 2:** Laboratory data of patient groups with and without mortality due to acute aortic dissection.

Variables	Mortality (+)	Mortality (-)	*P*-value
	n = 30	n = 83	
White blood cell (10³/ µL)	15.8 ± 9.1	10.3 ± 8.6	**0.001**
Platelet count (10³/ µL)	268 ± 91	256 ± 82	0.583
Hemoglobin (g/dL)	12.3 ± 1.4	12.2 ± 1.4	0.921
Admission plasma glucose (mg/dL)	86 ± 18	90 ± 17	0.250
Admission creatinine (mg/dL)	1.67 ± 1.21	0.94 ± 0.45	**0.003**
Blood urea nitrogen (mg/dL)	92.9 ± 84.2	44.4 ± 30.4	**0.005**
eGFR (mL/min/1.73 m2)	63.4 ± 35.3	89.6 ± 21.9	**0.001**
Thyroid stimulating hormone (mIU/L)	2.13 ± 1.11	2.36 ± 0.91	0.089
Albumin (g/dl)	3.80 ± 0.29	3.82 ± 0.26	0.595
Total protein (g/dL)	6.89 ± 0.34	6.91 ± 0.22	0.620
High-sensitivity C-reactive protein (mg/L)	40.7 ± 27.1	12.7 ± 18.9	**< 0.001**
Sodium (mmol/L)	139 ± 6.3	137 ± 5.9	0.176
Potassium (mmol/L)	4.47 ± 0.60	4.37 ± 0.56	0.089
Uric acid (mg/dL)	3.87 ± 0.45	3.88 ± 0.43	0.846
Triglycerides (mg/dL)	156 ± 49	153 ± 84	0.868
High-density lipoprotein (mg/dL)	48 ± 10	49 ± 11	0.632
Low-density lipoprotein (mg/dL)	102 ± 25	121 ± 31	**0.003**
Alanine aminotransferase (u/L)	18 ± 11	19 ± 13	0.815
Aspartate aminotransferase (u/L)	23 ± 15	24 ± 14	0.972
Calcium (mg/dL)	8.8 ± 0.30	8.7 ± 0.39	0.086
Peak troponin T level (ng/mL)	89 ± 161	86 ± 206	0.932
Peak creatine kinase-myocardial band (U/L)	23 ± 24	20 ± 32	0.722
N-terminal pro-brain natriuretic peptide (pg/mL)	2050 ± 2619	555 ± 1405	**0.005**
Left ventricular ejection fraction, (%)	55.2 ± 6.6	58.1 ± 5.2	**0.014**
Total follow-up duration (day)	9.73 ± 10.5	13.1 ± 6.6	0.071

eGFR=estimated glomerular filtration rate

**Table 3:** Medical treatment data of patient groups with and without mortality due to acute aortic dissection.

Variables	Mortality (+)	Mortality (-)	*P*-value
	n = 30	n = 83	
ACEi/ARB/ARNI use, n (%)	9 (30%)	55 (66%)	**0.001**
Beta-blocker use, n (%)	10 (33%)	43 (52%)	0.092
Acetylsalicylic acid use, n (%)	9 (30%)	43 (52%)	0.064
Statin use, n (%)	8 (27%)	41 (49%)	**0.025**
Calcium canal blocker use, n (%)	4 (13%)	12 (14%)	0.978

ACEi=angiotensin-converting enzyme inhibitors; ARB=angiotensin receptor blockers; ARNI=angiotensin receptor-neprilysin inhibitor

### Identification of Parameters that Independently Determine Patients with Mortality

Multivariable logistic regression analysis was performed to determine the parameters closely and independently associated with mortality. As a result of this analysis, it was found that age, CHA_2_DS_2_-VA score, and hs-CRP serum level independently determined the patients with mortality (Table 4). Among these parameters, each one unit increase in age, CHA_2_DS_2_-VA, and hs-CRP levels predicted 11.3%, 2.19-fold, and 3.2% increase in mortality in patients with AAD, respectively (Table 4).

**Table 4:** Multivariate logistic regression analysis to identify acute aortic dissection patients with mortality.

	Odds	95% Confidence interval	*P*-value
Age (each year)	1.112	1.067 - 1.232	0.004
CHA_2_DS_2_-VA score (each 1)	2.192	1.290 - 4.407	0.002
High-sensitivity C-reactive protein (each 1 mg/L)	1.032	1.009 - 1.057	0.001

### Receiver Operating Characteristic Curve Analysis of CHA_2_DS_2_-VA Score as a Predictor of Mortality

**Fig. 1:**
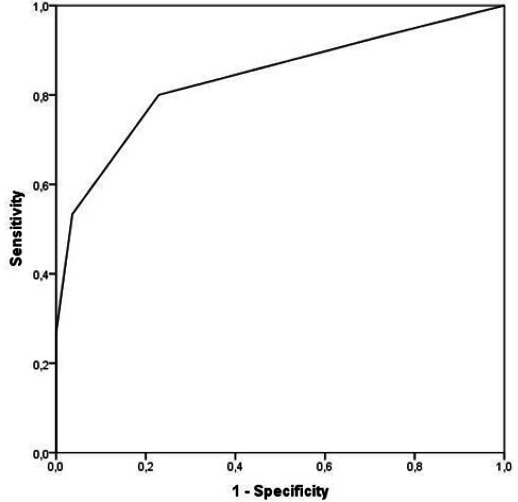
Receiver operating characteristic curve for CHA_2_DS_2_-VA score for identifying patients with mortality.

When ROC curve analysis was performed for the CHA_2_DS_2_-VA score to determine mortality, it was found that the CHA_2_DS_2_-VA score independently determined mortality, and the area under the ROC curve was 0.838 (*P* < 0.001 and 95% confidence interval 0.743 - 0.933) (Figure 1). When the cutoff value of CHA_2_DS_2_-VA score was taken as 3, it was found to determine the development of mortality due to AAD with 80.5% sensitivity and 78.6% specificity.

## DISCUSSION

The main findings of this study can be summarized as follows: (1) CHA_2_DS_2_-VA score is independently associated with the development of in-hospital mortality in patients with AAD and this finding was shown for the first time in the literature; (2) despite improvements in the diagnosis and treatment of AAD, in-hospital mortality is still 27.5%; (3) CHA_2_DS_2_-VA score, with a cutoff value ≥ 3, predicts in-hospital mortality due to AAD with acceptable sensitivity and specificity; (4) in addition to CHA_2_DS_2_-VA score, age and hs-CRP parameters are predictive of in-hospital mortality in patients with AAD. This finding is consistent with previous studies in the literature.

Despite advances in diagnosis and treatment, AAD is still a disease with high in-hospital mortality (15 - 30%)^[18,20]^. Therefore, it may be important to identify patients with mortality risk in advance and to plan follow-up and treatment accordingly. Many demographic, clinical, laboratory, and perioperative conditions have been shown to be associated with in-hospital mortality in patients with AAD^[19]^. These include advanced age^[19,29]^, male sex^[29]^, DM^[21]^, increased body mass index^[30]^, increased C-reactive protein, troponin, NT-proBNP, D-dimer, and white blood cell count^[19,23,31,32]^. In a recent systematic review and meta-analysis by Zhang Yi et al.^[19]^, 23 studies and 5,510 patients were evaluated, and it was shown that advanced age, male sex, presence of shock, malperfusion findings, and cardiac tamponade were independently associated with AAD in-hospital mortality. Consistent with the previous metanalysis, in our study, age, white blood cell count, hs-CRP, and NT-proBNP levels, and the presence of shock and malperfusion were found to be higher in patients with mortality.

The CHA_2_DS_2_-VA score is a scoring system mainly used to determine the risk of thromboembolism development in patients with AF and to identify patients to be given anticoagulant therapy^[1]^.

Anticoagulant therapy is recommended for patients with CHA_2_DS_2_-VA score ≥ 2 due to the high risk of thromboembolism. Apart from its importance in stroke detection, CHA_2_DS_2_-VASc score has been shown to be a mortality predictor in CV diseases^[2-13]^. Among the factors included in the CHA_2_DS_2_-VA score, HF, HT, advanced age, presence of stroke, and vascular disease have been shown to be associated with mortality in AAD cases^[13,18-25]^. In the presence of DM, there are conflicting results regarding both AAD development and AAD in-hospital mortality^[21,26,27]^. Although there are data suggesting that AAD development and mortality are less in patients with DM^[26,27]^, there are also data suggesting that DM itself is associated with in-hospital mortality because it is associated with perioperative mortality^[21]^. In our study, only 12 (10.6%) patients with AAD had DM, and the presence of DM was not associated with mortality. AAD is more common in men, and the presence of male sex is independently associated with mortality^[18-20]^. In our study, we used the CHA_2_DS_2_-VA score instead of the CHA_2_DS_2_-VASc score to determine in-hospital mortality in AAD, unlike previous studies, both because the latest AF guidelines removed sex from the risk score and because male sex has been shown to be associated with mortality in AAD cases. In our study, the frequency of male patients was higher in patients with AAD in accordance with the literature, but there was no independent relationship between male sex and mortality development.

It is clear that risk factors other than DM, which include CHA_2_DS_2_-VA score, are all individually associated with AAD mortality^[13,18-25]^. As we found in our study, the development of hypotension and shock is associated with mortality in patients with AAD^[19]^. Patients with HF are more susceptible to this clinical condition. NT-proBNP levels are high in HF patients. In a study, it was shown that high NT-proBNP level was associated with in-hospital mortality in patients with AAD^[31]^. In our study, NT-proBNP level was higher and LVEF level was lower in the patient group with AAD-related mortality. However, these two parameters were not independently associated with AAD mortality in multivariable analysis. HT increases aortic wall stress and is an important risk factor for the occurrence of AAD, the spread of dysplasia, and increased in-hospital mortality^[13,18,20]^. Effective blood pressure control is one of the most important steps in the treatment of patients with AAD until the operation. Age is one of the most important determinants of mortality in patients with AAD. Many studies and meta-analyses have shown that advanced age is independently associated with AAD mortality^[19,22-25]^. Especially in patients aged ≥ 80 years, AAD mortality is two-fold higher^[22]^. Similarly, age is the most important determinant of the CHA_2_DS_2_-VA score, with ≥ 75 years receiving a double score. In our study, in accordance with previous studies and literature, it was determined that the patients with mortality due to AAD were older, and age was an independent parameter in determining mortality. In the CHA_2_DS_2_-VA score, the other risk factors that scored two points other than age were stroke and peripheral thromboembolism. As in our study, cerebral malperfusion has been shown to be associated with mortality in patients with AAD^[19]^. The last parameter of the CHA_2_DS_2_-VA score is the history of vascular disease and patients who already have AAD score 1. AAD is more common in patients with previous vascular disease, large aorta, and aortic surgery^[18,20]^. In patients with previous stroke and limited cerebral perfusion such as carotid artery stenosis, new AAD may increase cerebral hypoperfusion and lead to increased mortality. In our study, it was shown that patients with a history of stroke had more mortality due to AAD, but this parameter was not an independent determinant. The last parameter of the CHA_2_DS_2_-VA score is vascular disease and directly includes AAD. The presence of concomitant peripheral arterial disease and aortic plaque in AAD patients may increase the prognosis of these patients. In conclusion, except for DM, all parameters in the CHA_2_DS_2_-VA score are directly related to AAD prognosis. Although the presence of DM is not directly associated with AAD prognosis, it is an important risk factor in the development of in-hospital mortality for perioperative medium-high surgical procedures^[33]^. Therefore, it may be appropriate to include this parameter in the CHA_2_DS_2_-VA score in the prognosis evaluation of patients.

### Limitations

This study had several important limitations. The study was single-center and retrospective. In our study, only in-hospital mortality was analyzed, and morbidity was not evaluated. Advanced renal failure, stroke, etc. due to AAD occur frequently. Smoking is a major CV risk score. There is also an association between smoking and AAD mortality^[18,20,34]^. Unfortunately, smoking is not included in the CHA_2_DS_2_-VA score, and this is a limitation for this score to determine the AAD mortality. This is an important limitation not only for us but also for previous studies that have shown an association between CHA_2_DS_2_-VASc score and CV mortality. Another limitation of the CHA_2_DS_2_-VA score in patients with AAD is the inverse association between DM and the occurrence of AAD and mortality. As previously mentioned, DM may be a perioperative risk factor and a prognose parameter in this group of patients. A recent study demonstrated that elevated serum lactate levels independently predict perioperative mortality in patients with AAD^[35]^. In our study, however, serum lactate levels could not be evaluated as a predictor of mortality because lactate measurements were not available for all patients. Various risk scores, such as the European System for Cardiac Operative Risk Evaluation, Society of Thoracic Surgeons score, and the specific German Registry of Type A AAD score, are commonly used to assess perioperative risk in cardiac surgery. In our study, we did not perform a comparison between these scoring systems and the CHA_2_DS_2_-VA score in terms of their prognostic value for AAD-related mortality. Such an analysis might have provided additional insights. Therefore, the routine use of the non-specific CHA_2_DS_2_-VA score in cardiac surgical procedures cannot be recommended, unlike the disease-specific scoring systems.

## CONCLUSION

The CHA_2_DS_2_-VA score is independently associated with the development of in-hospital mortality in patients with AAD. The close prognostic association of the CHA_2_DS_2_-VA score with CV diseases is due to the fact that all of its parameters are associated with adverse CV events. While this scoring system was initially known and used by AF-related specialties, it is now widely known by all physicians. Therefore, it can be used not only for the prevention of thromboembolic complications in AF but also as a general prognosis parameter, including AAD.

## Data Availability

The authors declare that the data supporting the findings of this study are available within the article.

## Artificial Intelligence Usage

The authors declare that no artificial intelligence tool was used in the preparation of this article.
